# A Case Report of Whipple’s Disease: A Challenging Diagnosis

**DOI:** 10.7759/cureus.41021

**Published:** 2023-06-27

**Authors:** Filipa Nunes, Martim Trovão Bastos, Maria João Fernandes, Jéssica Oliveira, Mariana Costa

**Affiliations:** 1 Internal Medicine Department, Hospital Prof. Doutor Fernando da Fonseca, Lisbon, PRT

**Keywords:** weight loss, whipple disease, tropheryma whipplei, bilateral pleural effusion, constitutional syndrome

## Abstract

Whipple’s disease is caused by a ubiquitous Gram-positive bacillus, *Tropheryma whipplei*. The disease is extremely rare, with only 1,000 cases reported worldwide. Classic Whipple’s disease is characterized by a multisystemic involvement with joint (arthralgias) and gastrointestinal (abdominal pain, diarrhea, and weight loss) symptoms.

We present a case of a 48-year-old male who had a constitutional syndrome associated with an exuberant bilateral pleural effusion. The small bowel biopsy identified a rod-shaped bacterial cologne in the macrophage cytoplasm, positive for periodic acid-Schiff (PAS) staining, and the polymerase chain reaction (PCR) exam identified the DNA of *Tropheryma whipplei*. The patient was medicated with two weeks of endovenous antibiotherapy with ceftriaxone 2 g per day, followed by one year of oral trimethoprim 160 mg and sulfamethoxazole 800 mg twice daily. He presented good evolution with total resolution of symptoms.

## Introduction

Whipple’s disease is caused by a ubiquitous Gram-positive bacillus, *Tropheryma whipplei*. The disease is extremely rare, with only 1,000 cases reported worldwide [1]. The spectrum of the disease is wide. Classic Whipple’s disease is characterized by a multisystemic involvement with joint (arthralgias) and gastrointestinal (abdominal pain, diarrhea, and weight loss) symptoms [2]. Usually, articular symptoms precede the others by many years [3]. When isolated, it affects predominantly the central nervous system or the cardiovascular system (heart valves) [4]. The authors present a challenging diagnosis of Whipple’s disease.

## Case presentation

A 48-year-old Romanian male presented with weight loss (approximately 10 kilograms), anorexia, and dyspnea for minor efforts, for the last three months. These symptoms impeded him from working as a construction worker. The patient denied arthralgias, nausea, vomiting, diarrhea, or blood loss. He did not have any recent travel or exposition to animals. He did not take any medication and reported a previous medical history of alcoholism (70 grams of alcohol per day) and tabagism (80 pack-year units).

**Figure 1:**
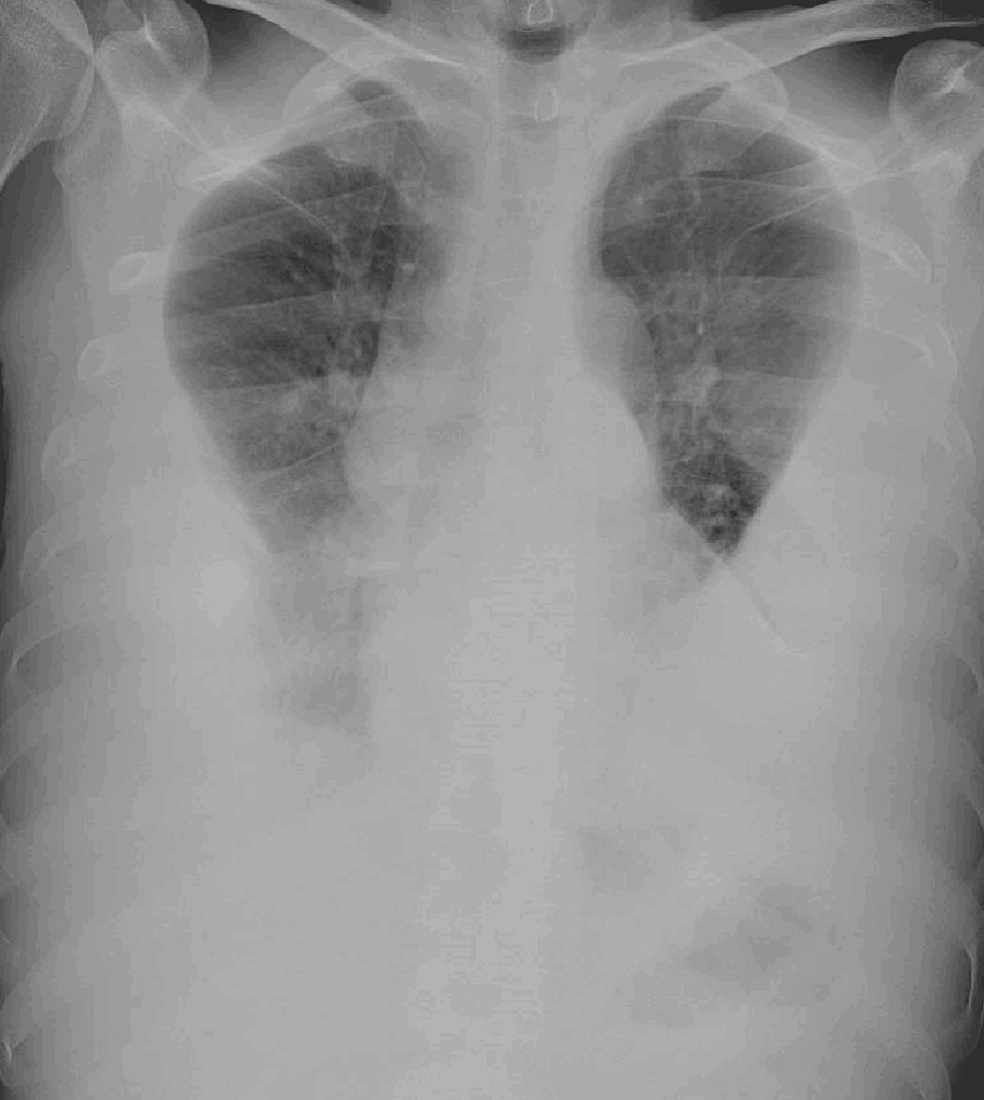
Extensive bilateral pleural effusion.

At admission, blood test results revealed normocytic and normochromic anemia, folate acid deficiency (2 ng/mL), and iron deficiency (iron 29 ug/dL, transferrin saturation 13%, total iron-binding capacity 177 ug/dL, ferritin 511 ng/mL, and RDW 15.1). He did not have leukocytosis (6.3x10^9/L) or neutrophilia (4.5x10^9/L). C-reactive protein and erythrocyte sedimentation rate were elevated at 5.22 mg/dL and 91 mm, respectively. The autoimmune study revealed positive antinuclear antibodies titer of 1:160. Urine sample was normal. The viral serologies for HIV and hepatitis were negative. The chest x-ray revealed bilateral increased density and loss of both costophrenic angles compatible with extensive pleural effusion (Figure 1).

**Figure 2:**
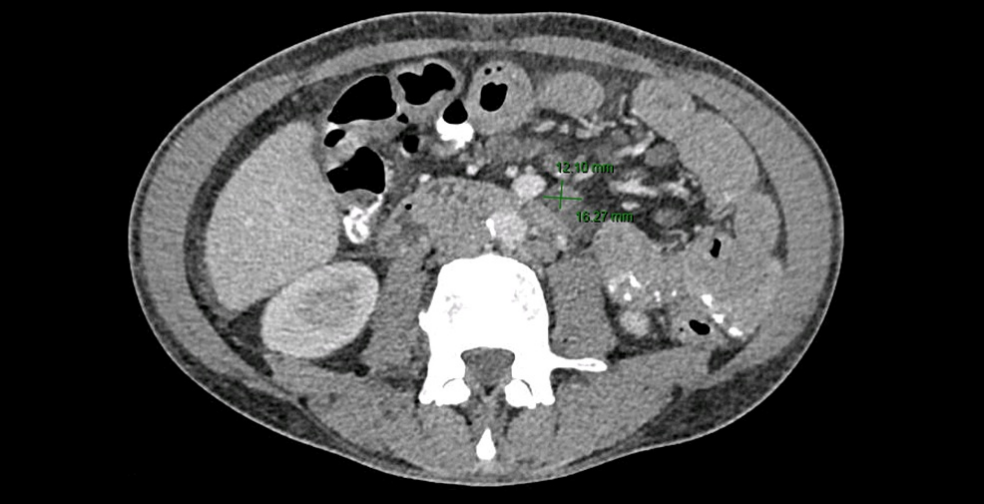
Multiple adenopathies in the root of the mesentery (the biggest with 16x12 mm).

The patient was admitted to the internal medicine department for the investigation of weight loss associated with severe bilateral pleural effusion and anemia. A thoracentesis was performed, draining 1,800 mL of serous fluid, compatible with transudate. There were no neoplastic cells, and the cultural exam of the pleural fluid was negative. The full-body CT scan revealed multiple adenopathies in the root of the mesentery (the biggest with 16x12 mm), and its nature was imprecise in this exam, but surveillance was recommended. There were no other relevant alterations in this exam (Figure 2). 

**Figure 3:**
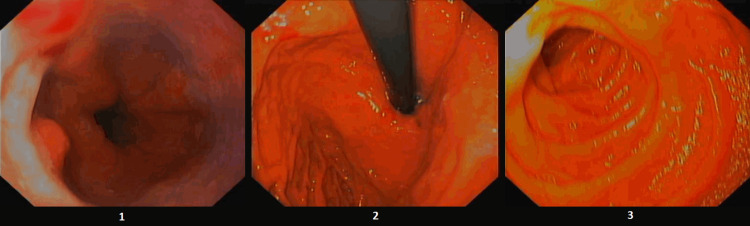
Grade A esophagitis and erosive gastropathy. 1 - Gastroesophageal junction with non-confluent erosions < 5 cm. 2 - Fundus with an erythematous mucosa and scattered erosions. 3 - Duodenum D2 with no lesions.

To complete the study of occult neoplasm, an upper gastrointestinal (GI) endoscopy was performed. The upper GI endoscopy showed grade A esophagitis and erosive gastropathy (Figure 3).

**Figure 4:**
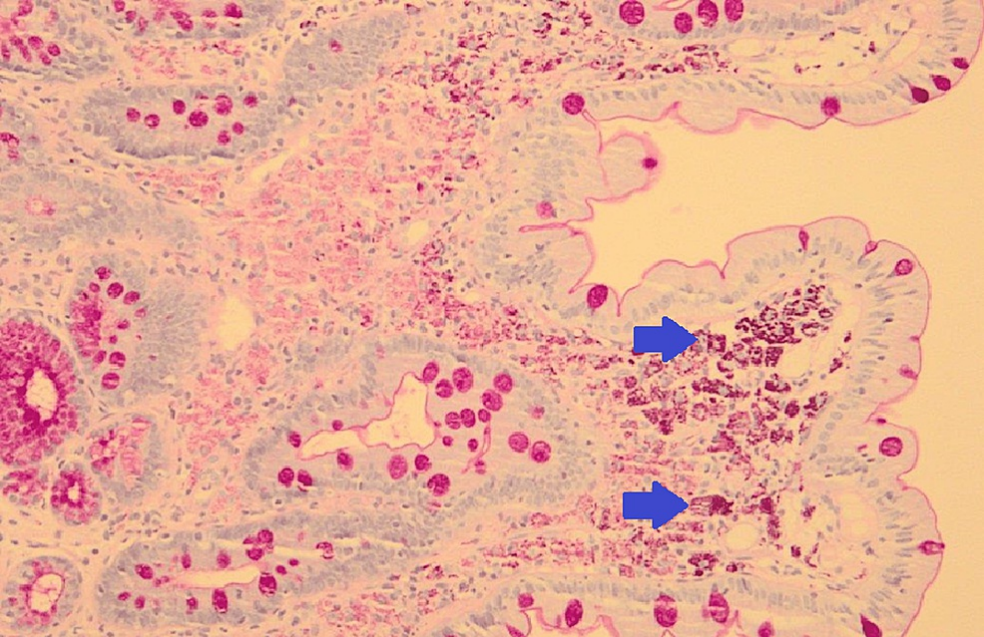
Rod-shaped bacterial colony in the macrophage cytoplasm, PAS-positive (blue arrows).

The duodenal and gastric biopsies revealed active chronic duodenitis and chronic non-atrophic gastritis. The histochemical analysis demonstrated a rod-shaped bacterial colony in the macrophage cytoplasm, positive for periodic acid-Schiff (PAS) staining (Figure 4).

The polymerase chain reaction (PCR) exam identified the DNA of *Tropheryma whipplei*, and the diagnosis of Whipple’s disease was made. Cerebrospinal fluid examination excluded neurological involvement. Cardiac involvement of the disease was excluded by transesophageal echocardiogram.

The patient was medicated with two weeks of endovenous antibiotherapy with ceftriaxone 2 g per day followed by one year of oral trimethoprim 160 mg and sulfamethoxazole 800 mg twice daily. The patient maintained follow-up in the internal medicine consultation and presented good evolution with total resolution of symptoms. After two years of follow-up, the patient is asymptomatic with no recrudescence of the symptoms.

## Discussion

Diagnosing Whipple's disease remains challenging as the symptoms are nonspecific and vague. It usually takes from three to seven years after the beginning of symptoms and the final diagnosis due to the low clinical suspicion of the diagnosis [5]. The spectrum of the disease is wide. Classic Whipple’s disease is characterized by arthralgias, weight loss, diarrhea, and abdominal pain. This classic presentation associated with a constitutional syndrome leads to the investigation and exclusion of neoplastic causes in the first approach. A hallmark of the disease is that joint involvement usually precedes the other symptoms by many years, and the arthralgias are migrant and involve large peripheral joints, such as the knees, wrists, and ankles. Sometimes, classic Whipple’s disease is misdiagnosed as seronegative inflammatory arthritis. Thus, seronegative rheumatoid arthritis that does not improve with therapy should raise suspicion for Whipple’s disease [6]. Gastrointestinal symptoms usually present with abdominal pain and diarrhea (watery diarrhea and steatorrhea). This leads to severe wasting syndrome, which results in severe weight loss due to malabsorption.

Other features of the disease include systemic involvement with lymphadenopathy (mesenteric and mediastinic), which affects half of the patients with this disease; neurological involvement, which usually occurs in the latter stages of the disease, presenting as dementia, nystagmus, or myoclonus; cardiac involvement, which is extremely rare and may present as endocarditis, pericarditis, or myocarditis; skin involvement related to the malabsorption of vitamins, which may present as skin hyperpigmentation; and pulmonary involvement courses with pleuropulmonary affection, such as pleural effusions, chronic cough, interstitial-lung disease-like presentation, and pulmonary hypertension [4].

The diagnosis is made by microorganism identification. The diagnostic criteria include identification of PAS-positive foamy macrophages in the small bowel biopsy, PCR detection of *Tropheryma whipplei*, and immunohistochemical staining with *Tropheryma whipplei* antibodies. Other laboratory findings include anemia, leukocytosis, and neutrophilia. Iron, folate, and B12 vitamin deficiency are also prevalent [7].

Treatment relies on antibiotic therapy. The standard of care is two weeks of ceftriaxone, followed by trimethoprim 160 mg and sulfamethoxazole 800 mg twice daily for 12 months [7].

The prognosis of Whipple's disease depends on several factors, including the stage of the disease at the time of diagnosis and the promptness of treatment. Early recognition and treatment generally lead to better outcomes. With appropriate and timely treatment, most patients experience significant improvement in their symptoms and have a good prognosis. Regular follow-up and monitoring are necessary to manage the disease and prevent relapses.

## Conclusions

This clinical case illustrates the challenge of diagnosing Whipple’s disease. The clinical case made us suspect a neoplastic etiology causing the patient's symptoms. The workup consisted in ruling out a solid neoplasm with the full body CT scan, thoracentesis, and endoscopic exams. With the neoplastic hypothesis becoming less probable, an infectious etiology was evoked. In this scenario, a small bowel biopsy was paramount for the diagnosis since the patient did not have any localizing symptoms, and it made the diagnosis of Whipple’s disease.

Although a rare condition, Whipple’s disease should be suspected in the cases of constitutional symptoms with multisystemic affection, especially when there is the involvement of joints and the gastrointestinal system and after ruling out the hypothesis of neoplasm. The disease should also be suspected in cases of seronegative rheumatoid arthritis that does not improve with therapy. Early recognition of Whipple's disease is crucial for initiating timely treatment, relieving symptoms, preventing long-term complications, and ensuring proper surveillance and follow-up care. Whipple's disease is a complex disorder, and there are still several areas, such as pathogenesis, biomarkers, genetic factors, long-term outcomes, and management, that require further study to enhance our understanding of the condition. Collaborative efforts among researchers, clinicians, and patient groups are crucial to conduct studies and advance the knowledge of Whipple's disease. 
